# Improvement of Left Ventricular Ejection Time Measurement in the Impedance Cardiography Combined with the Reflection Photoplethysmography

**DOI:** 10.3390/s18093036

**Published:** 2018-09-11

**Authors:** Shing-Hong Liu, Jia-Jung Wang, Chun-Hung Su, Da-Chuan Cheng

**Affiliations:** 1 Department of Computer Science and Information Engineering, Chaoyang University of Technology, Taichung 413, Taiwan; shliu@cyut.edu.tw; 2 Department of Biomedical Engineering, I-Shou University, Kaohsiung 824, Taiwan; 3 Institute of Medicine, School of Medicine, Chung-Shan Medical University, Taichung 402, Taiwan; 4 Department of Internal Medicine, Chung-Shan Medical University Hospital, Taichung 402, Taiwan; 5 Department of Biomedical Imaging and Radiological Science, China Medical University, Taichung 404, Taiwan; dccheng@mail.cmu.edu.tw

**Keywords:** stroke volume, left ventricular ejection time, impedance cardiography, photoplethysmography

## Abstract

Cardiac stroke volume (SV) is an essential hemodynamic indicator that can be used to assess whether the pump function of the heart is normal. Non-invasive SV measurement is currently performed using the impedance cardiography (ICG). In this technology, left ventricular ejection time (LVET) is an important parameter which can be determined from the ICG signals. However, the ICG signals are inherently susceptible to artificial noise interference, which leads to an inaccurate LVET measurement and then yields an error in the calculation of SV. Therefore, the goal of the study was to measure LVETs using both the transmission and reflection photoplethysmography (PPG), and to assess whether the measured LVET was more accurate by the PPG signal than the ICG signal. The LVET measured by the phonocardiography (PCG) was used as the standard for comparing with those by the ICG and PPG. The study recruited ten subjects whose LVETs were simultaneously measured by the ICG using four electrodes, the reflection PPG using neck sensors (PPG_neck_) and the transmission PPG using finger sensors (PPG_finger_). In each subject, ten LVETs were obtained from ten heartbeats selected properly from one-minute recording. The differences of the measured LVETs between the PCG and one of the ICG, PPG_neck_ and PPG_finger_ were −68.2 ± 148.6 ms, 4.8 ± 86.5 ms and −7.0 ± 107.5 ms, respectively. As compared with the PCG, both the ICG and PPG_finger_ underestimated but the PPG_neck_ overestimated the LVETs. Furthermore, the measured LVET by the PPG_neck_ was the closest to that by the PCG. Therefore, the PPG_neck_ may be employed to improve the LVET measurement in applying the ICG for continuous monitoring of SV in clinical settings.

## 1. Introduction

Cardiogenic shock refers to a group of syndromes in which heart function is extremely decreased, resulting in a significant reduction in cardiac output and causing severe acute peripheral circulatory failure. Consequently, cardiogenic shock is the ultimate manifestation of heart pump failure. When the heart pump is insufficient, it cannot maintain its minimum cardiac output, leading to a drop in blood pressure. Seriously insufficient blood supply to vital organs and tissues can lead to systemic circulatory dysfunction. Thus, a series of pathological and physiological processes characterized by ischemia, hypoxia, metabolic disorders, and vital organ damages will emerge as a result [1,2]. In traditional emergency rooms or intensive care units, in order to understand the condition of the heart for patients with heart failure, the patient may be equipped with a Swan-Ganz catheter. This Swan-Ganz catheter (also called pulmonary artery or right heart catheter) inserted into a pulmonary artery can provide important hemodynamic data to diagnose heart failure or sepsis, monitor therapy, and estimate the drug effects. Through the thermal dilution, cardiac hemodynamic parameters such as stroke volume (SV) and cardiac output can be measured intermittently and continually [3]. The thermal dilution (also called thermo dilution) is a method of cardiac output determination, in which a bolus of solution (usually saline) of known temperature and volume is injected into the right atrium, and the resultant change in blood temperature is sensed by a thermistor previously arranged in the pulmonary artery with a catheter. However, this method requires surgery to insert the catheter into the body, so its application may be strictly limited.

Kubicek et al. have proposed a non-invasive measurement technique to measure the cardiac hemodynamic parameters using the impedance cardiography (ICG) method [4]. High-frequency current signals are input through the surface electrodes to detect changes in the capacitive impedance of the thoracic cavity caused by heart beats in order to assess the SV [5]. In recent years, cardiac fluid dynamics parameter monitoring research using the ICG technology has been applied in the anesthesia monitoring [6,7], the pediatric intensive care unit monitoring [8], and the status assessment of increase in exercise load [9,10]. The advent of these studies is mainly based on the fact that the ICG technology is fundamentally non-invasive and convenient.

Left ventricular ejection time (LVET) is an important parameter in the SV determination with the ICG technology. As we known, detection of the time difference between the first and the second heart sound in the heart sound signal is a standard method for the LVET measurement. In the LVET measurement with the ICG technique, the ICG waveform is first differentiated and the time difference is determined between the first zero crossing point and the lowest point in the differential ICG signal. However, both low signal-to-noise ratio and body motion-induced interference noise frequently make the ICG signal instable, which in turn causes a decrease in the accuracy and preciseness of the LVET measurement. Further, this leads to a big error in the assessed SV. In recent years, arterial blood pressure waves have been derived through the PPG method. The PPG method is mainly divided into two types: one is transmission and the other is reflection [11]. The transmission PPG is mainly used for measuring peripheral arterial pulse waves at the places such as fingers or ears, while the reflection PPG can measure arterial pulse waves at any part of the body, such as wrists, foreheads or necks. 

The purpose of this study was to evaluate whether the measured LVET at different locations using the PPG technique was more reliable than that by the ICG technique, and to see whether the transmission PPG or the reflection PPG could produce more accurate LVETs as compared with the phonocardiography (PCG). In the study, ten healthy adult males were included to simultaneously perform four kinds of LVET measurement. The results showed that the LVET estimated by using the PPG technique was more accurate and stable than the ICG technique. LVETs measured from the arteries close to the heart by the transmission PPG were more stable than those measured from the peripheral arteries by the reflection PPG.

## 2. Materials and Methods

### 2.1. Hardware Architecture

**Figure 1:**
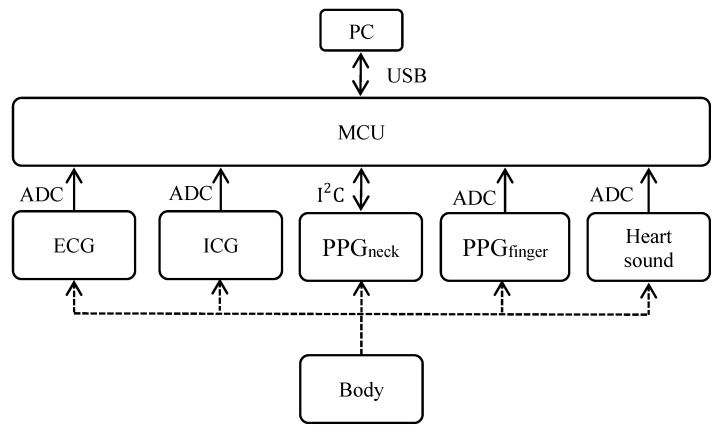
Hardware architecture of this study for physiological signal acquisition.

The hardware circuit diagram of this study for physiological signal acquisition is shown in Figure 1. Several human physiological signals are simultaneously recorded in the study, including the electrocardiogram (ECG) signal, ICG, PPG_neck_ (reflection PPG sensor placed on the neck), PPG_finger_ (transmission PPG sensor placed on the finger), and PCG signals. The PPG_neck_ sensor was embedded in a digital chip (ADPD174, Analog Devices, Norwood, MA, USA) and it communicated with a microcontroller unit (MCU) (STM32L152VBT6, STMicroelectronics, Plymouth, MN, USA) by means of the I^2^C interface. The analog to digital conversion (ADC) for the other signals was processed by the MCU. The resolution of ADC was 12 bits, and the sampling frequency was 500 Hz. After the signal conversion was completed, the MCU transmitted these signals to a computer for display and storage through a USB port.

**Figure 2:**
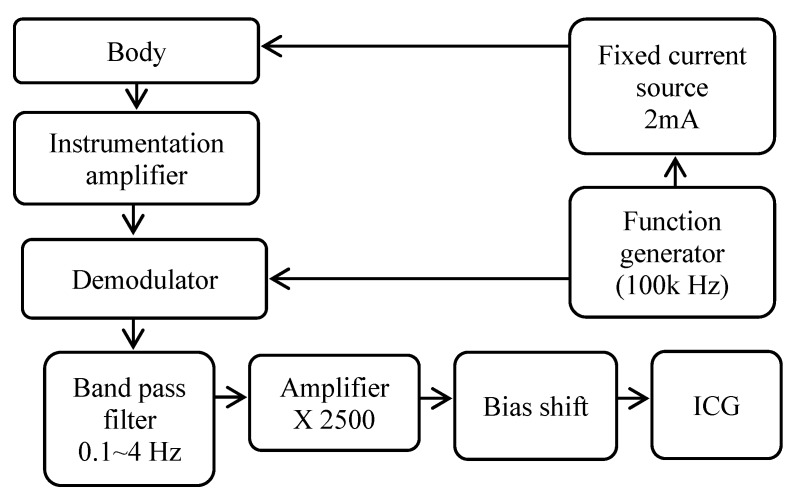
ICG signal circuit architecture.

Figure 2 shows the ICG signal measurement circuit architecture. The architecture is similar to that in Qu et al.’s study [8]. First, a radio frequency (RF) signal running through a constant current source of 2 mA was delivered to the human body. The RF signal with a frequency of 100 kHz was generated by a programmable waveform generator (AD9837, Analog Devices, Norwood, MA, USA). An instrumentation amplifier (AD8422, Analog Devices) was used to pick up the modulated signal from the human body. The modulated signal was demodulated to obtain the original ICG signal by a multiplier chip (AD835, Analog Devices). The original ICG signal had very low amplitude and was always contaminated with large noise. To improve the signal-to-noise ratio of the ICG signal, the original signal was first passed through a band-pass filter (0.1 Hz~4 Hz), and amplified by an amplifier with a gain of 2500. In the end stage, the level boost circuit was employed to raise the bias of the ICG signal to 1.5 V.

### 2.2. Arrangement of Placement of Sensing Elements

**Figure 3:**
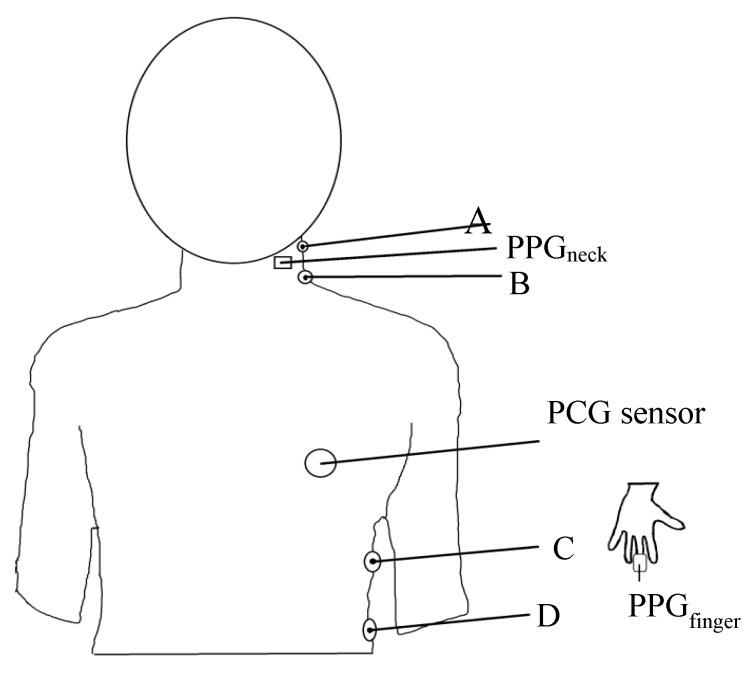
Arrangement and placement of sensing elements on the human body.

The electrodes of the measurement system and the placement of the PPG sensors are shown in Figure 3. In the ICG measurement, the RF signal produced by the constant current source enters into the human body through one pair of electrodes (A and D). Temporarily, through the other pair of electrodes (B and C), the instrumentation amplifier is used to pick up the ICG signal as well as the ECG signal. The PPG_neck_ sensor is placed on the left side of the neck, the PPG_finger_ sensor is clamped to the finger of subject’s left hand, and the PCG sensor is placed over the chest and near the heart.

### 2.3. Stroke Volume Measurement

**Figure 4:**
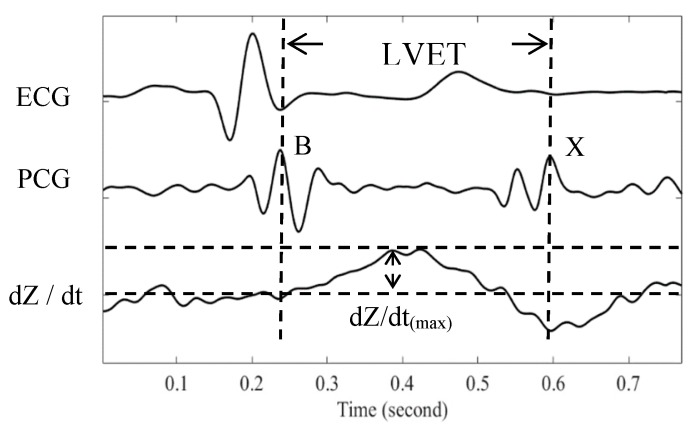
Relationship among the ECG, PCG and d*Z*/d*t* signals.

From the ICG signal, cardiac SV can be calculated by the following equation proposed by Kubicek [4]:

(1) $$SV=rho_{b}\cdot {\left(\frac{L}{Z_{0}}\right)}^{2}\cdot LVET\cdot dZ/dt_{\left(\max\right)}$$

where *rho_b_* is the blood impedance that is assumed to be a constant value of 150 ohm-cm, *L* is the distance between the B and C electrodes, *Z*_0_ is the impedance of thoracic cavity, and d*Z*/d*t*_(max)_ is the maximum change of the ICG impedance signal. According to Equation (1), the SV has an absolute linear relationship with the LVET. Figure 4 shows the relationship among the ECG signal, the PCG signal and the d*Z*/d*t* of ICG signal. The B and X points in the heart sound signals represent the opening and closure of the aortic valve, respectively. The time difference between these two points (B and X) is defined as the LVET. In the differential ICG signal, d*Z*/d*t*, the LVET is defined the time duration between the first zero crossing point and the minimum point.

In the PCG signals, the B and X points are the time points of the maximum peak of the first and second heart sound, respectively. The LVET is determined as the time duration between the B and X points. Therefore, the LVET is initiated by the opening of the aortic valve, and is terminated by the closing of the aortic valve. During the LVET, the blood is actually pumped from the left ventricle to the aorta. Thus, in the blood pressure waveform, the point B represents the time for the first zero crossing point of its differential waveform and the point X the time for the first maximum valley of its differential waveform. Moreover, the point X should occur just before the dicrotic notch, and the location of the dicrotic notch may be used to estimate the point X. According to the ICG theory, the B and X points in the differential ICG waveform are used to determine the LVET, as show in Figure 4 [4,5,12]. It is known that the arterial blood pressure waveform can be reconstructed using the PPG signals [2]. Therefore, the same points in the PPG signals were also used to determine the LVET in this study.

### 2.4. Experimental Protocol

The experiment in this study was divided into three steps, including the collection of experimental data, data processing and analysis, and statistical analysis. This study recruited ten healthy male subjects with no cardiac diseases or injured limbs. Their age was between 22 and 27 years (23.5 ± 1.7 years), weight was between 49 and 96 Kg (66.0 ± 13.1 Kg), and height was between 163 and 185 cm (172.6 ± 5.8 cm). This experiment was approved by the Research Ethics Committee of China Medical University & Hospital (No. CMUH107-REC3-061), Taichung, Taiwan.

The measurement duration for each subject was one minute. During the measurement, ECG, PCG, ICG, PPG_finger_, and PPG_neck_ signals were measured at the same time. After the signals were filtered by the 20 Hz third-order Butterworth low-pass filter and the 0.2 Hz first-order Butterworth high-pass filter, the best 10 heartbeat signals for each subject were properly selected by visual inspection. The 10 LVETs corresponding to the 10 heartbeats in the PCG signal were uncovered and used as the standard values. Furthermore, LVETs corresponding to the 10 heartbeats in the ICG and PPG signals were detected, respectively. Finally, the means and the standard deviations of the difference between the PCG-measured LVET and the other techniques-measured LVET (i.e., ICG, PPG_finger_ and PPG_neck_) were used to investigate their accuracy and stability.

## 3. Results

**Figure 5:**
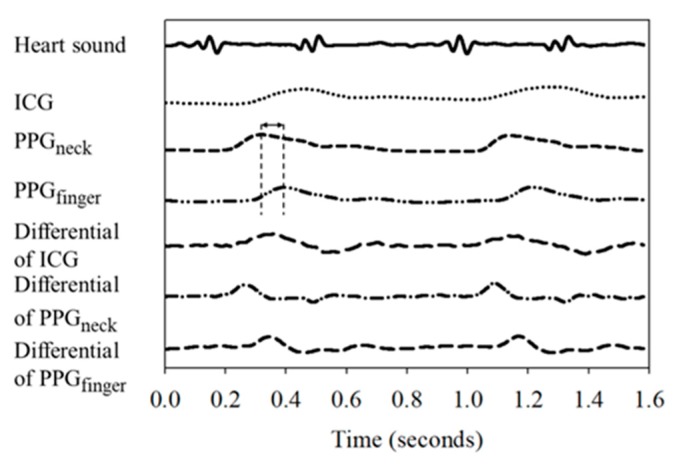
Timing relationship between the PCG, ICG, PPG and their differential signals.

Typical physiological signals measured from one subject in this study are shown in Figure 5, including the heart sound (solid line), ICG (dotted line), PPG_neck_ (short dash line), PPG_finger_ (dash-dot-dot line), and their differential signals, ICG (long dash line), PPG_finger_ (dash-dot line), and PPG_neck_ (medium dash line). It can be seen that both PPG signals are more stable than the ICG signal, and there is a significant delay of about 78 ms between the PPG_finger_ signal and the PPG_neck_ signal.

**Figure 6:**
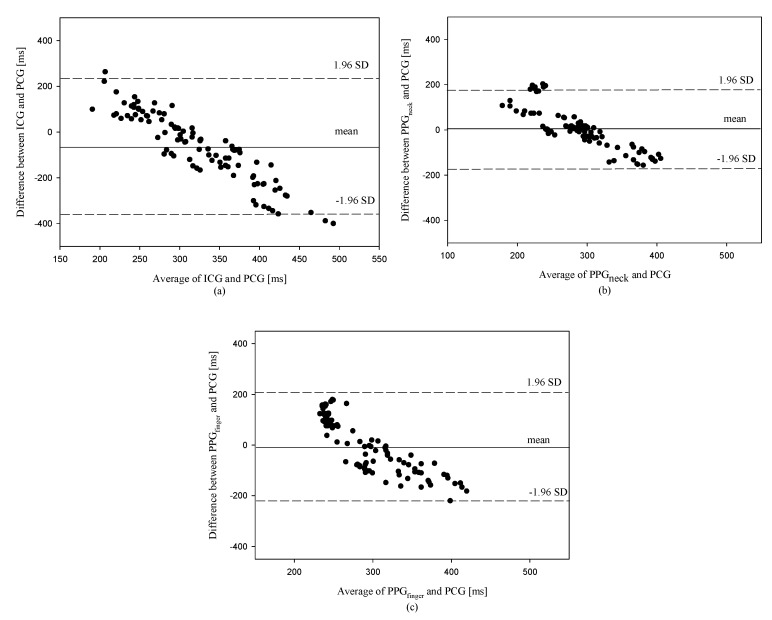
Bland and Altman plots of the correspondent differences of the LVET measured by the PCG technique and the other techniques, ICG, PPG_neck_ and PPG_finger_, (**a**) PCG and ICG techniques, (**b**) PCG and PPG_neck_ techniques, (**c**) PCG and PPG_finger_ techniques.

For each subject, 10 heartbeats were selected from the ICG, PCG, PPG_finger_, and PPG_neck_ signals. Table 1 shows the measured LVETs with the four techniques. Table 2 shows the correlation coefficients, *r*^2^, between the standard LVET measured by the PCG signal and the three LVETs measured by the ICG, PPG_neck_, and PPG_finger_ signals for the ten subjects. The correlation coefficients were 0.178 ± 0.162, 0.270 ± 0.199 and 0.129 ± 0.149 for the ICG, PPG_neck_, and PPG_finger_ signals, respectively. The PPG_neck_ technique had a highest correlation coefficient, 0.680, for subject 5. The ICG technique had a worst correlation coefficient, 2.31 × 10^−6^, for subject 4. Table 3 shows that the corresponding differences between the LVETs measured by one of the three techniques (ICG, PPG_neck_ and PPG_finger_) and the LVETs measured by the PCG technique are −68.2 ± 148.6 ms, 4.8 ± 86.5ms and −7.0 ± 107.5 ms. In brief, both the ICG and PPG_finger_ underestimated but the PPG_neck_ overestimated the LVETs as compared with the PCG. Besides, it can be seen that the LVETs measured by the PPG_neck_ were the closest to those measured by the PCG. In addition, the measured LVETs were significantly smaller with the ICG than the PCG (*p* < 0.01), but the measured LVETs with the PPG_neck_ and PPG_finger_ did not significantly differ from those with the PCG (*p* = 0.58; *p* = 0.51). Figure 6 shows Bland and Altman plots of the correspondent differences of the LVET measured by the PCG technique and the other techniques, ICG, PPG_neck_ and PPG_finger_. We found that the larger the LVET measured by the PCG, the more the underestimated amount of the LVET measured by the ICG and PPG_finger_. The accuracy of LVET measurement is the lowest for the ICG technique, as shown in Figure 6a. Its corresponding difference has the largest bias shift and SD, −68.2 ms and 148.6 ms, respectively. Some data are beyond the ±1.96 SD. Although the differences of some LVETs measured by the PCG and PPG_neck_ techniques are beyond the −1.96 SD, as shown in Figure 6b, the SD (86.5 ms) of the corresponding difference is the lowest, as compared with other techniques.

## 4. Discussion

The ICG technology was developed about half a century ago [4]. But, the medical instrument of ICG has been employed in the clinical monitor or diagnosis since a decade ago. Cybulski et al. have reviewed the published papers listed in Web of Science during the period of 2011–2012 [12], and found 99 published papers relating to “impedance cardiography.” They have also pointed out that the application fields of the ICG include not only the cardiac hemodynamic parameter monitor, but also the posture change for the heart stress [9,13], the cardiac rehabilitation [14], the optimal settings for the pacemakers [15], and the obstructive sleep apnea [16,17]. In those previous studies, the LVET is always considered as one of physiological important parameters. The ICG, fundamentally, is a noninvasive technique, and its clinical operation is really safer and easier than the application of the invasive Swan Ganz or PiCCO^®^ technique.

**Figure 7:**
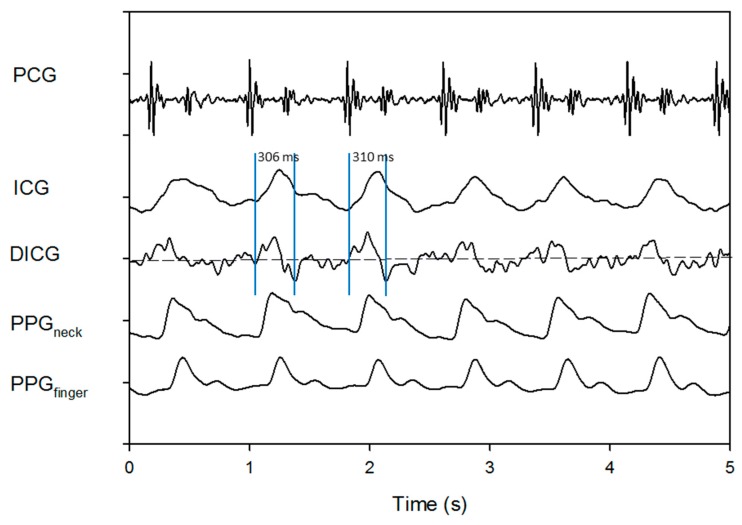
Heart sound, stable ICG signal, its differential signal (DICG), PPG_neck_ and PPG_finger_.

**Figure 8:**
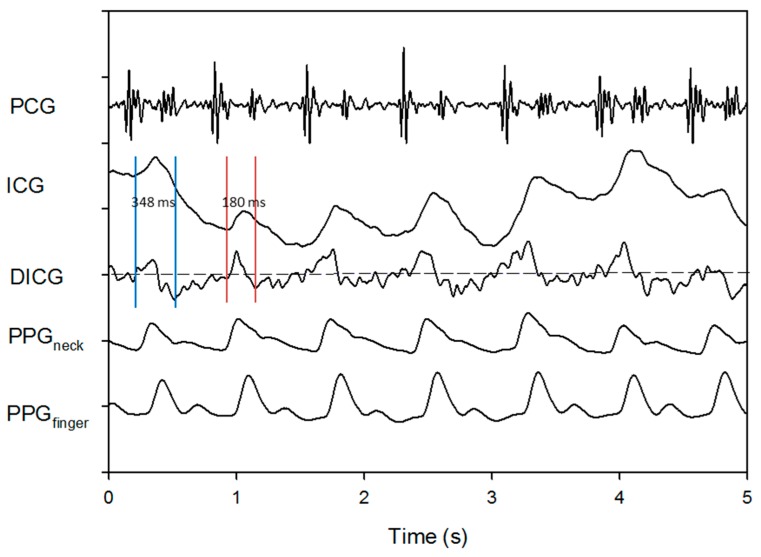
Heart sound, unstable ICG signal, its differential signal (DICG), PPG_neck_ and PPG_finger_.

It is usually difficult to obtain a stable ICG signal, since during the ICG measurement the tissue impedance is vulnerable to a variety of body motions, such as the body shaking, breathing and other human disturbances [18]. Moreover, the ICG signal represents the relatively little change in the liquid volume inside the chest cavity. So, the amplitude of the measured electrical ICG signals is usually minute and merely about 10–50 μV. The frequency of the baseline driftin the ICG signal would be equivalent to that of the respiration or body shaking. As we known, only a stable ICG signal can be used to yield a reliable LVET. Unfortunately, the baseline drifts of the ICG signal mainly caused by the body motions do consequently lead to an unstable differential waveform of the ICG signal, and then provide an inaccurate LVET estimation. Figure 7 shows a stable ICG signal, in which two LVET values from two adjacent heartbeats are estimated to be 306 and 310 ms, respectively. Due to high stability and good quality of such ICG signal, the two estimated LVET values are pretty close. On the contrary, with a slightly unstable ICG signal (Figure 8), two LVET values from two adjacent heartbeats are assessed to be 348 and 180 ms, respectively. It is clear that a big difference exists between the two LVET values. Obviously, it is the baseline drift that makes the ICG signal unstable. Therefore, according to our study, the two PPG signals, PPG_neck_ and PPG_finger_, are found to be more stable than the ICG signal. As shown in Figure 8, there is a more serious baseline drift in the ICG signal, compared with that in either of the PPG signals. This is one of the major reasons that the PPG signals are utilized to estimate LVET value in the present study.

**Figure 9:**
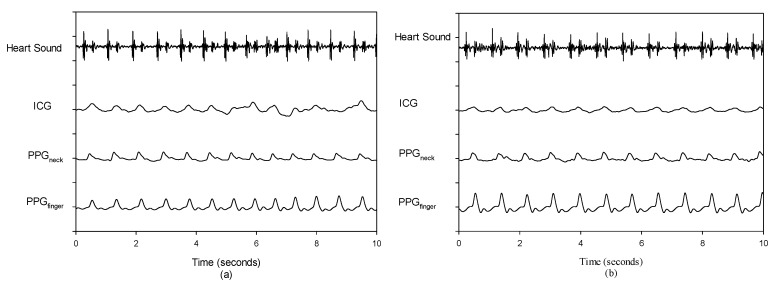
Time series signals recorded by using the PCG, ICG, PPG_neck_, and PPG_finger_: (**a**) Subject 5, (**b**) Subject 4.

In our experiments, we find that the PPG signal appears more stable than the ICG signal. Therefore, ten-beat segments from the PPG_neck_ signal in individual subject are properly selected to estimate the LVET. Then, the same ten-beat segments from the ICG and PPG_finger_ signals are used to determine the LVET. A best correlation coefficient (0.68) exists between the LVETs measured by the PPG_neck_ and PCG techniques in Subject 5. On the contrary, the correlation coefficient of the LVET measured by the ICG and PCG technique is only 0.153 in that subject. Figure 9a shows four time series signals of the PCG, ICG, PPG_neck_ and PPG_finger_ for Subject 5, in which there is an obvious dicrotic notch point on individual heartbeat in the PPG_neck_ signal. But, the dicrotic notch point in the ICG signal only shows up on some heartbeats. Figure 9b shows the signals by the PCG, ICG, PPG_neck_, and PPG_finger_ for Subject 4, in which the dicrotic notch point does not appear in the ICG signal. As a result, the correlation coefficient of his LVET measured by the ICG and PCG techniques is pretty low (only 2.31 × 10^−6^). Although the PPG_finger_ signal usually shows the clear dicrotic notch points, these dicrotic notch waves possess serious distortion, and their happening time can be easily affected by the presence of hydrostatic pressure in large arteries or vena cava, and the vascular characteristics.

The second issue is about why the measured LVETs with the reflection PPG sensor have more accurate than those with the transmission PPG sensor, as compared with the standard (that is the LVETs determined by the PCG). This is because the transmission PPG sensor is put on the fingers during the measurement, and the blood vessels in the fingers are remote to the heart and belong to the peripheral circulation. As blood is pumped from the heart, it will be delivered to the periphery after several times of bifurcations and conversions. The peripheral arterial blood pressure waves are really affected by the characteristics of the arterial blood vessels along the delivering path. Thus, alteration and transformation in the peripheral pressure waveform finally results in inaccurate LVET assessment. In the reflection PPG, when the sensor are positioned at some place close to the aorta (e.g., the neck), the measured PPG signal seems less affected by the characteristics of the conduit arteries from the heart to the neck. Nevertheless, the present result shows that the measured LVETs with the reflection PPG are more accurate than those with the transmission PPG. Furthermore, a relatively small difference of 4.8 ± 86.5 ms exists between the LVETs measured by using the PCG and the PPG_neck_, with a relatively large difference of −7.0 ± 107.5 ms by using the PCG and the PP_finger_ in the study. Also, with the ICG, the measured LVET is found to be far from that with the PCG, with a big difference of −68.2 ± 148.6 ms.

LVET measurement with the PCG signalshas been considered as the standard method [3]. The sensor used to pick up the PCG signal usually is a piezoelectric element, or a microphone (a capacitive or inductive transducer) [3]. To yield a stable PCG signal with low-level noise, the sensor must be placed on a precise position over the chest. However, in our experimental experience, it is hard for an operator to achieve a suitable position for the PCG sensor in a short time. Furthermore, the size of the PCG sensor is large, and a driving circuit needed to drive the sensor must be designed by the users themselves. However, the PPG sensor and its related driving circuits have been integrated into a chip, like as ADPD174GGI of Analog Devices [19] or MAX30101 of Maxim Integrated [20]. Due to their small size and low power consumption, these chip-type PPG sensors have been widely used in wearable device applications. By the way, the PPG signal is more easily measured than the PCG signal. It is the reason, in the study, that the PPG sensors are selected to measure the PPG signals and then to estimate the LVET values for the SV measurement with the ICG technology.

According to the LVET measurement with the arterial pressure signal, the time of the arterial pressure beginning to rise is the start time for the LVET, whereas the time of the arterial pressure commencing to fast decline is the end time for the LVET.In previous studies, most investigators have applied this criterion in the ICG signal to detect the LVET [4,5,12]. Therefore, in the study we use the same principle in the ICG and PPG signals to estimate the LVET. Unfortunately, we find that the estimated LVET values by the ICG and PPG signals become inaccurate as compared with the PCG-based LVET values when they are larger and lower than 300 ms, as shown in Figure 6. The primary systolic time interval consists of the pre-ejection period (PEP), LVET and electromechanical systole. The PEP is the total duration of the electrical and mechanical events prior to ejection. The LVET is initiated by the opening of the aortic valve, following the end point of PEP [21]. The LVET estimated by the ICG or PPG signals may be affected by the PEP. The PEP basically correlates with the velocity and Frank Levinson index of contractile element [22], and the heart rate [21]. Therefore, it is possible in the study that the variability in the LVET estimation by the ICG or PPG may be partially caused by the PEP.

## 5. Conclusions

This paper proposes two concepts to improve the accuracy in calculating cardiac SV with the ICG technique, in which PPG_neck_ signal is used to determine the LVET. Through the experiments, compared with the LVET by the standard method, the LVET measured by the PPG_neck_ signal is indeed more accurate than that by the ICG. Secondly, the closer the artery detected by the PPG sensor is to the heart, the more accurate the measured LVET will be. Although the LVETs measured by PPG_neck_ signals only has a moderate correlation with those by the PCG signals, and still has a large variability for the correspondent difference with the PCG signals, they appear more accurate and higher correlation than those measured by the ICG signals. In the future, according to the results of this study, the reflection PPG sensors may be utilized in the ICG instrument to enhance the stability and accuracy of the LVET measurement in clinical settings.

## Figures and Tables

**Table 1:** The LVETs measured by the PCG, ICG, PPG_neck_, and PPG_finger_ for the ten subjects.

Subject No.	PCG (ms)	ICG (ms)	PPG_neck_ (ms)	PPG_finger_ (ms)
1	337.8 ± 96.1	286.8 ± 12.4	376.2 ± 81.4	293.8 ± 10.8
2	413 ± 46.7	439.4 ± 26	453.4 ± 45.6	331.2 ± 5.1
3	506.8 ± 109.9	246.8 ± 9.9	339.6 ± 13	242.2 ± 2.4
4	351.4 ± 92.3	275.8 ± 6.8	204.2 ± 7	283.2 ± 2.5
5	338 ± 90.6	286.4 ± 7.7	300.2 ± 97.6	300 ± 7
6	193.8 ± 86	137.8 ± 7.4	161.4 ± 2.3	321.4 ± 10.8
7	199.2 ± 25.4	422.4 ± 32.9	193.6 ± 29.2	296.8 ± 23.1
8	506.8 ± 132.6	322.2 ± 14.4	305.2 ± 119.1	285.8 ± 6.9
9	452 ± 117.3	278.2 ± 37.9	309 ± 18.3	302.4 ± 9.3
10	288.2 ± 98.2	162 ± 25.3	332.6 ± 45.9	248.6 ± 11.1

**Table 2:** The correlation coefficients, *r*^2^, between the standard LVET measured by PCG signal and the three LVETs measured by ICG, PPG_neck_, and PPG_finger_ signals for ten subjects.

Subjects No.	ICG	PPG_neck_	PPG_finger_
1	0.001	0.435	0.070
2	0.493	0.028	0.001
3	0.004	0.131	0.017
4	2.31 × 10^−6^	0.309	0.002
5	0.153	0.680	0.342
6	0.065	0.042	0.025
7	0.281	0.187	0.350
8	0.240	0.101	0.359
9	0.384	0.347	0.124
10	0.161	0.441	0.001
All	0.178 ± 0.162	0.270 ± 0.199	0.129 ± 0.149

**Table 3:** The correspondent differences between the LVETs measured by one of the three techniques (ICG, PPG_neck_ and PPG_finger_) and the LVETs measured by PCG technique for ten subjects.

Subjects No.	ICG (ms)	PPG_neck_ (ms)	PPG_finger_ (ms)
1	−44.0 ± 97.0	7.0 ± 9.7	−82.4 ± 79.2
2	−81.8 ± 43.3	−108.2 ± 25.7	−122.2 ± 45.7
3	−264.6 ± 110.0	−4.6 ± 11.0	−97.4 ± 12.9
4	−68.2 ± 92.4	7.4 ± 5.8	79.0 ± 7.5
5	−38.0 ± 93.5	13.6 ± 4.4	−0.2 ± 93.7
6	127.6 ± 83.9	183.6 ± 11.8	160.0 ± 11.4
7	97.6 ± 23.6	−125.6 ± 31.0	103.2 ± 46.7
8	−221.0 ± 129.4	−36.4 ± 13.9	−19.4 ± 123.4
9	−149.6 ± 123.3	24.2 ± 33.3	−6.6 ± 17.3
10	−39.6 ± 103.2	86.6 ± 19.8	−84.0 ± 47.6
All	−68.2 ± 148.6	4.8 ± 86.5	−7.0 ± 107.5
